# Deep-Learning-Based Segmentation of Cells and Analysis (DL-SCAN)

**DOI:** 10.3390/biom14111348

**Published:** 2024-10-23

**Authors:** Alok Bhattarai, Jan Meyer, Laura Petersilie, Syed I. Shah, Louis A. Neu, Christine R. Rose, Ghanim Ullah

**Affiliations:** 1 Department of Physics, University of South Florida, Tampa, FL 33647, USA; alokb@usf.edu (A.B.); syedislamudd@usf.edu (S.I.S.); 2 Institute of Neurobiology, Faculty of Mathematics and Natural Sciences, Heinrich-Heine-University, 40225 Düsseldorf, Germany; jan.meyer.11@hhu.de (J.M.); laura.petersilie@hhu.de (L.P.); losch115@hhu.de (L.A.N.); rose@uni-duesseldorf.de (C.R.R.)

**Keywords:** live cell imaging, cell segmentation, tracking ion dynamics, streamlit, tracking morphological changes

## Abstract

**Highlights:**

- A python-based tool called DL-SCAN for automated cell segmentation and thorough analysis of fluorescence microscopy image data.
- A user-friendly interface with several pre- and post-processing options to streamline the analysis workflow.
- Single- and multi-cell analysis options.
- DL-SCAN provides valid segmentation results on both synthetic and real biological data.
- DL-SCAN tracks changes in cells’ morphology over time in response to pathological events such as ischemia.
- DL-SCAN generates a range of statistics, such as rise time, rise rate, decay time, decay rate, duration, and amplitude of time traces with a few clicks.

**Abstract:**

With the recent surge in the development of highly selective probes, fluorescence microscopy has become one of the most widely used approaches to studying cellular properties and signaling in living cells and tissues. Traditionally, microscopy image analysis heavily relies on manufacturer-supplied software, which often demands extensive training and lacks automation capabilities for handling diverse datasets. A critical challenge arises if the fluorophores employed exhibit low brightness and a low signal-to-noise ratio (SNR). Consequently, manual intervention may become a necessity, introducing variability in the analysis outcomes even for identical samples when analyzed by different users. This leads to the incorporation of blinded analysis, which ensures that the outcome is free from user bias to a certain extent but is extremely time-consuming. To overcome these issues, we developed a tool called DL-SCAN that automatically segments and analyzes fluorophore-stained regions of interest such as cell bodies in fluorescence microscopy images using deep learning. We demonstrate the program’s ability to automate cell identification and study cellular ion dynamics using synthetic image stacks with varying SNR. This is followed by its application to experimental Na^+^ and Ca^2+^ imaging data from neurons and astrocytes in mouse brain tissue slices exposed to transient chemical ischemia. The results from DL-SCAN are consistent, reproducible, and free from user bias, allowing efficient and rapid analysis of experimental data in an objective manner. The open-source nature of the tool also provides room for modification and extension to analyze other forms of microscopy images specific to the dynamics of different ions in other cell types.

## 1. Introduction

Recent advancements in imaging technology for studying biological processes at scales ranging from a single molecule to the cell network and tissue level have revolutionized biological research. In particular, fluorescence microscopy techniques are widely used to study the structure and/or function of cells, or spatiotemporal changes thereof, under a variety of conditions [1,2,3,4,5,6]. These experiments generate huge image-based datasets, which may have many different objects (e.g., molecules, organelles, and cells) that need to be analyzed. Typically, this kind of analysis relies heavily on manual or semi-automated approaches, which are labor intensive, costly, not very accurate, and often poorly reproducible. Thus, fully automated methods that can overcome these issues are key to utilizing the full potential of these powerful experimental techniques.

An additional problem is the dynamic nature of the biological processes under study, which makes the analysis of imaging data even more cumbersome. For example, in many organs such as the brain, the concentration of the molecules of interest, e.g., of ions such as Ca^2+^ and Na^+^, undergoes transient changes both in time and space [7,8,9], while tissue movements additionally shift the exact location of the cells or cellular compartments in the field of view. In such a scenario, the user is often required to draw or adjust the regions of interest (ROIs) in which fluorescence signals are to be analyzed frame by frame. In extreme conditions, such as ischemia and seizures, brain cells swell significantly, causing the tissue to move both on the x–y and the *z*-axis [10,11]. In those experiments, proper analysis of fluorescence signals is even more time-consuming. This makes the use of accurate automated methods even more desirable.

Chemical fluorescent indicators or genetically encoded sensors are routinely used to study the spatial and/or temporal dynamics of various ions including Ca^2+^ and Na^+^. The fluorescence emission of these fluorophores can be examined using different microscopy techniques after being introduced into the sample [12]. Microscope vendors have developed several tools to serve this purpose (e.g., NIS Elements 6.0 from Nikon Europe B.V., Amstelven, the Netherlands, or Cell Sense FluoView 3.1.1 from Evident Europe GmbH, Hamburg, Germany). These tools mainly rely on manual selection of ROIs, and additional programs for data evaluation are often required to assess the images and experimental datasets in more detail. For imaging of changes in intracellular Ca^2+^, for example, several tools have recently emerged (e.g., AQuA, CaSCaDe, MSparkles) that automatically select ROIs based on changes in the intensity of neighboring pixels [13,14,15,16]. Most of these tools were developed with a specific application in mind, such as dynamic Ca^2+^ imaging in vivo. In addition, many of them rely on the use of MATLAB commercial toolboxes and require extensive knowledge of MATLAB as well as prior coding experience.

To overcome the above-mentioned drawbacks, we have developed a tool called DL-SCAN. DL-SCAN is based on Python and is available as open-source software. One of the development goals of DL-SCAN was to ensure usability without extensive training or prior knowledge of Python as a programming language. This is achieved through a simplified user experience with a browser-based interface. DL-SCAN takes microscopy image time series data as an input, applies a deep learning algorithm to segment fluorescently labeled objects and then extracts statistics about the dynamics of ions under normal and pathological states. A range of options for pre-processing and post-processing are also provided. The automated nature of DL-SCAN is designed to mitigate bias and reduce the analysis time, ensuring verifiable and reproducible results. Furthermore, we posit that the development of open-source tools, such as ours, will be beneficial in the study of ion dynamics under various physiological and pathological conditions. Finally, the open-source and user-friendly nature of our approach makes extending and customizing our program for other experimental conditions or needs straightforward.

## 2. Materials and Methods

To develop an automated tool that pre-processes, segments, and extracts various properties of time-lapse microscopy image stacks (in TIFF format, but other formats can be easily added), we made use of Python 3.8.8 and a python library called Streamlit (version 1.21.0). This allowed the development of a clean and user-friendly graphical user interface (GUI) without HTML, CSS, and JS. Here, we outline the key characteristics of DL-SCAN.

### 2.1. Algorithm and Key Features of the Application

This section aims to provide a general overview of the features of the tool, while each feature is thoroughly detailed with screenshots in the User Manual (Supplementary Information Text). DL-SCAN initiates within a browser as a local host upon following the steps as mentioned in the User Manual (Supplementary Information Text). With “Pre-processing and Segmentation” being the homepage of the tool, it allows the user to load a time-lapse microscopy image stack. As soon as the file is loaded, the program checks whether the pixel values are already in 8-bit unsigned integer format. If not, it performs the conversion and, subsequently, the page displays each image frame of the original stack as selected by the user. To speed up the loading and the following processes, initial conversion to 8-bit unsigned integer format before loading is recommended. Depending on the quality of the images generated due to variations in the experimental setup, various pre-processing techniques might be necessary to segment, characterize, and analyze them with a high level of accuracy. This tool provides multiple pre-processing options to the user that include background correction, Gaussian blur, median blur, brightness and contrast adjustments and Contrast Limited Adaptive Histogram Equalization (CLAHE).

Background subtraction is often required to remove signals not generated by the fluorophores being analyzed, such as tissue autofluorescence. Upon selecting “Background Correction”, the user can draw a single rectangle on the first image frame and select a region apparently free of distinct cellular fluorescence. At the backend, the code then computes the average intensity of the pixels in the selected background region and subtracts it from each pixel value across all frames to achieve the desired correction. The background-corrected frames will be displayed immediately after region selection. It should be noted that these background-corrected images are not used to segment the cells, but to extract their intensity profiles to aid robust analysis. The processed frames as a result of user-selected preprocessing options are then displayed.

To improve accuracy for robust segmentation, DL-SCAN uses maximum intensity projection (MIP) along each line of the image stack exclusively for segmentation (e.g., see analysis performed for experiments shown in Figure 4). Therefore, it is important to note that the ROIs should possess higher intensity values than the background when this option is used. This process ensures that all ROIs are properly identified and segmented once the deep learning segmentation algorithm is applied. The resulting collapsed image is then displayed and is now ready for segmentation. Notably, instead of the collapsed image, users can also choose to use the first image in the stack for segmentation (e.g., see analysis performed for experiments shown in Figure 5).

Once the “Segment and Generate Labels” button is clicked, DL-SCAN applies a deep learning algorithm to identify and segment the objects. The segmented and labeled objects are then displayed. When proceeding with analysis, the tool performs a frame-by-frame analysis on every image of the stack, contingent upon the identification and segmentation of the objects.

StarDist is a convolutional neural network (CNN) deep learning architecture that works remarkably well in detection and segmentation of star-convex polygon shapes [17]. In other words, objects that exhibit visible boundaries from their centers are effectively predicted using StarDist [17]. The algorithm relies on U-Net based framework specifically designed to segment nuclei and cells in microscopy images, as the model is pre-trained on diverse fluorescent microscopy images of nuclei [18]. The model integrates object probability prediction and distances to the object boundary along a predetermined set of radial directions referred to as rays. The final result is achieved by applying non-maximum suppression (NMS), which is a technique to filter out overlapping or redundant object detections, determined by a set threshold. Given our focus on the detection and segmentation of neuronal and glial cells during the course of the tool development, we leveraged StarDist 0.8.3, which yielded favorable results without necessitating any modifications to the original training dataset.

Inaccurate segmentation results may stem from a low image signal-to-noise ratio, which can be addressed by applying various preprocessing techniques, for instance, using median blur to reduce salt-and-pepper noise, Gaussian blur to reduce Gaussian noise, adjusting brightness and contrast, applying background correction for autofluorescence removal, etc. In cases where the capabilities of the in-tool options fall short of a specific requirement, utilizing FIJI for a broader range of preprocessing options is recommended. At times, the pixel distribution in loaded images can be uneven due to differences in the experimental setup, resulting in overexposure in some areas and underexposure in others. To address this, we offer a Rolling Ball Background Correction (RBBC) option, which helps mitigate this problem. A ball of a user-selected radius rolls across the image, fitting into its valleys and crevices. The minimum pixel value within the region covered by the ball is subtracted from the pixel value at the center of the ball. By repeating this for each pixel of the image, it is effective at smoothing out the background when unevenly illuminated. In the instances where it is crucial, users also have the additional option to manually draw ROIs on the labeled collapsed image. These hand-drawn regions are automatically added to the list of labels for further analysis. Although this option provides users with flexibility in handling exceptional cases, it is not required by the software and should be avoided to ensure reproducibility and objectivity.

Upon cell detection, users can utilize the “Single-cell Analysis” tab located in the sidebar to explore the properties of each cell individually. To enhance accessibility and streamline referencing, the selected (either the first or the collapsed), segmented and labeled images from “Preprocessing and Segmentation” are once again presented within the “Single-cell Analysis” tab. Furthermore, downloadable and interactive tables are displayed separately, featuring mean intensity values and counts of bright pixel values surpassing a specific threshold for each identified cell across all frames. This threshold is user-adjustable and is referred to as the area threshold percentage. When segmented on the collapsed image, e.g., obtained from microscopy data for cells stained with cell-permeant dyes like Calcein or Fura-2 AM [19,20,21], its purpose is to account for the observed variations in cell areas across different frames, which effectively captures the phenomenon of spatial spread of the signal or cell swelling/shrinking with time.

Within the interactive table, users have the option to choose a single identified cell for in-depth analysis. Upon selecting the cell, an image with the highlighted chosen cell is presented, followed by a range of adjustable options. The “Frame Rate” widget enables users to input a value that converts frame numbers into time in seconds, based on the recording frequency in the experiment. Moreover, users can choose to conduct an analysis with or without bleaching correction (Supplementary Information Text), depending on the experimental setup and methodology employed. In the absence of bleaching correction, users are presented with choices to fine-tune the moving average for trace smoothing. This is followed by the option to select “Static” or “Dynamic” analysis. The “Single Frame Value” under the “Static” option allows users to input the baseline frame number (starting frame/reference frame), peak intensity frame number (frame of maximum activity), and signal recovery frame number (frame where the signal returns to normal). On the other hand, the “Average Frame Value” differs from the previous only in the sense that it asks users to select the number of consecutive frames to calculate their average intensity values as the baseline intensity. Here, frame refers to an individual, captured image within the stack that corresponds to a specific time point, and the choice of a single or average frame value depends entirely on the nature of the experiment.

With the “Dynamic” option selected, the program asks the user to select frame numbers for calculating baseline intensity using their mean intensity values, but the peak intensity and the recovery point are computed automatically by the algorithm. Subsequently, the normalized intensity table relative to the baseline, and various traces (Mean Intensity Vs. Time, Smoothed Mean Intensity Vs. Time, and Bright Pixel Area Vs. Time) are displayed. With bleaching correction enabled, several options remain consistent, as previously mentioned. Photobleaching, which refers to the gradual loss of fluorescence from a fluorophore over time due to light exposure, depends on the experimental conditions and is a common challenge in microscopy.

In our imaging experiments, we used a standard procedure to account for the specific experimental setup and the specific extent of photobleaching in each experiment and for each ROI. In more detail, we recorded baseline fluorescence without any additional experimental manipulation. Furthermore, after all manipulations (e.g., stimulation with glutamate) had been performed, we recorded the recovery of the induced signals back to baseline. Each of the resulting data traces was then normalized by calculating ΔF/F to account for differences in fluorescence intensity (dye concentration) between cells. Next, we fitted a mono-exponential decay of these traces, interpolating the data points between the initial baseline and recovery periods, and subtracted these fits from the traces on a point-by-point basis, resulting in a correction for photobleaching. In DL-SCAN, as is the case for the absence of a photobleaching correction option, the normalized intensity table in relation to the baseline, and various traces mentioned above, are displayed for the latter as well.

Further analysis is carried out on the “Smoothed Mean Intensity Vs. Time” trace to obtain some key parameters of changes in fluorescence intensity detected upon a specific experimental manipulation or stimulation in the chosen ROIs. These include the rise time, decay time, duration, rise rate, decay rate, and peak amplitude of the induced signals. Upon clicking the “Obtain the parameters for selected label” button, these values are presented in a downloadable table.

Within the “Multi-cell Analysis” tab located in the sidebar, the user has the ability to collectively analyze multiple cells. After selecting labels from the interactive table, traces for each selected cell are displayed. These traces can be toggled on and off by clicking the corresponding legend denoted by the respective label number. To isolate the trace of interest, a double-click action is required. Subsequently, the process employs all the options as outlined in the context of single-cell analysis. The resulting table, containing the properties of the chosen cells, is downloadable, making it available for further utilization. All these properties of the tool are comprehensively outlined in the User Manual (Supplementary Information Text).

### 2.2. Synthetic Data

**Figure 1:**
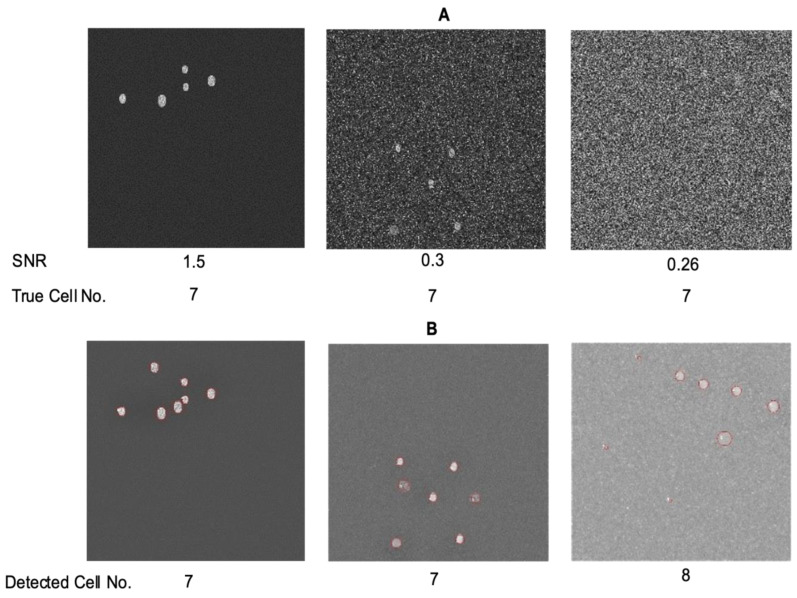
DL-SCAN can accurately detect cells in image stacks with different SNR values. First frames of synthetically generated images with a total of 7 cells (**A**) and DL-SCAN-identified cells on collapsed images (**B**) for varying SNR values.

**Figure 2:**
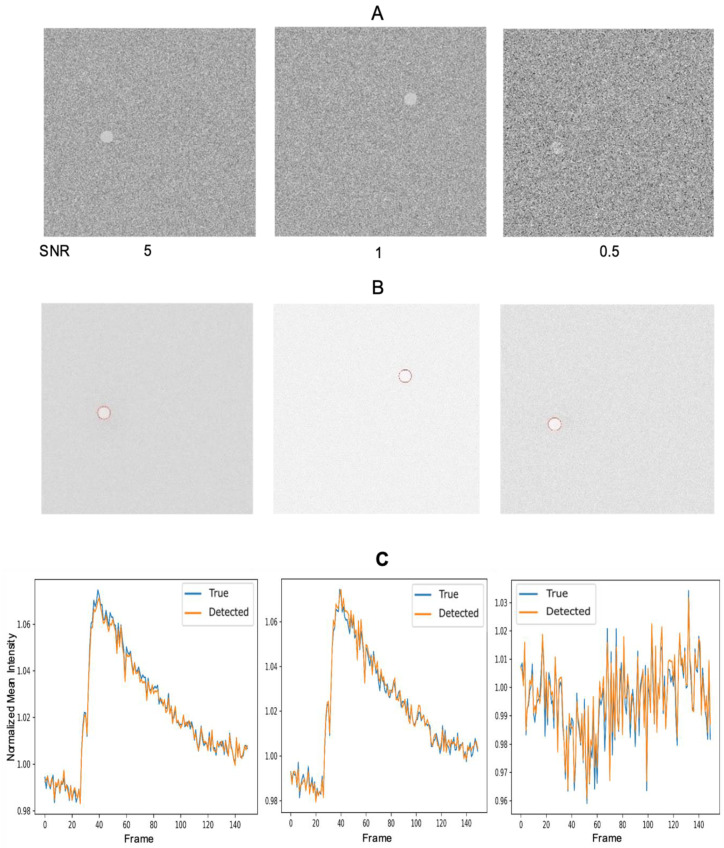
DL-SCAN can accurately extract time traces from images with different values of SNR. First frames of synthetically generated images with a single fixed cell across frames for varying SNR values (**A**) and DL-SCAN-identified cells on collapsed images (**B**). Comparison of the true normalized mean intensity with those extracted by DL-SCAN (**C**).

To test the effectiveness and robustness of the program before applying it to the real biological data, we first generated stacks of synthetic data (Figure 1 and Figure 2) resembling neuronal microscopy images (150 frames each in TIFF format) using Python 3.8.8. Single- and multiple-object datasets were produced across a range of SNR values. The datasets were created using a simple approach defined by the formula:

*F* (*W*, *H*, *t*) = *B* (*W*, *H*, *t*) + *O* (*x*, *y*, *t*) + *N* (*W*, *H*, *t*)

Here, *F* is the final image for the *t^th^* frame with width *W* and height *H* and is a result of the combination of the blank image (*B*), the objects added (*O*) at position (*x*, *y*), and the noise following normal distribution (*N*).

The SNR of the stack is then estimated as

$$SNR=\sqrt{\frac{{\sigma_{final}}^{2}}{{\sigma_{noise}}^{2}}-1}$$

where ${\sigma_{final}}^{2}$ and ${\sigma_{noise}}^{2}$ are the variances of the final image stack and the noise, respectively.

While each frame in the single-object datasets had one object, the multiple object datasets were constrained to include a total of 7 objects. Among these, 5 objects were consistently present from the 1^st^ to the 148^th^ frame, while an additional object was introduced in the last 2 frames at different locations. This setup was designed to demonstrate the program’s capability to detect cells that appear later in the image stack.

### 2.3. Experimental Methods

The previously published experimental study [9,22] used to test the validity of DL-SCAN was carried out in strict accordance with the institutional guidelines of the Heinrich Heine University Düsseldorf as well as the European Community Council Directive (2010/63/EU). All experiments using brain slices were communicated to and approved by the Animal Welfare Office at the Animal Care and Use Facility of the Heinrich Heine University Düsseldorf (institutional act no. O52/05). In accordance with the recommendations of the European Commission, animals up to 10 days old were killed by decapitation, while older mice were anesthetized with CO_2_ and then quickly decapitated. According to the German Welfare Act (TSchG; Section 4, paragraph 3), no additional approval was required for postmortem removal of brain tissue. No protected species were sampled.

The imaging experiments shown in Figure 4 and Figure 5 are taken from previously published work where experimental details and procedures are described in detail [9,22]. In brief, for the preparation of acute cortical or hippocampal tissue slices, BALB/c mice (both sexes) from postnatal day (P) 14–21 were anaesthetized, decapitated, and had their brains removed. The brains were then placed in ice-cold preparation saline (in mM: 130 NaCl, 2.5 KCl, 0.5 CaCl_2_, 6 MgCl_2_, 1.25 NaH_2_PO_4_, 26 NaHCO_3_, and 10 glucose) saturated with 95% O_2_/5% CO_2_ to achieve a pH of 7.4. Preparation saline was adjusted with high magnesium and low calcium to enhance slice viability by reducing neuronal excitation during the slicing process. Subsequently, brains were cut into 250 µm-thick slices using a vibratome (HM 650V, Thermo Fisher Scientific). For expression of the genetically encoded Ca^2+^ indicator GCaMP6f (“GCaMP”, ETH Zürich, Schweiz [23]), organotypic brain slice cultures were prepared from C57BL/6J male mouse cortex as described previously [24,25]. GCaMP was applied on the first three days after the preparation of slices using adeno-associated viral vector (AAV) delivery. Following this, transfected slices were kept in culture for at least ten more days for optimal sensor expression. Subsequently, organotypic brain slices were used for experiments at near physiological temperature (33 ± 1 °C) at a wide-field setup. For wide-field imaging, slices were kept for 20 min at 34°C in preparation saline containing the astrocyte-specific dye sulforhodamin 101 (SR101, 0.5–1 µM), followed by incubation for 10 min in artificial cerebrospinal fluid (ACSF, in mM: 130 NaCl, 2.5 KCl, 1.25 NaH_2_PO_4_, 26 NaHCO_3_, 2 CaCl_2_, 1 MgCl_2_, and 10 glucose, saturated with 95% O_2_/5% CO_2_, pH 7.4) at 34 °C. Slices used for multiphoton imaging were kept at 34 °C for 30 min in ACSF without SR101. Preparation saline and ACSF were both adjusted to an osmolarity of 310 mOsm l^−1^. Slices were kept in the dark in ACSF at room temperature (21 °C) for up to 6 h until use [22].

To measure changes in intracellular sodium ([Na^+^]_i_) or calcium ([Ca^2+^]_i_), slices were bolus loaded with the membrane-permeable form of the Na^+^-indicator ION-NaTRIUM-Green-2 (ING-2 AM; #2011F, Mobitec, Rheda-Wiedenbrück, Germany) or the Ca^2+^-indicator Oregon Green 488 BAPTA-1 (OGB-1-AM; #O6807, Invitrogen, Waltham, Massachusetts, USA). Astrocytes were identified by selective labelling with SR101 [26]. High-resolution multiphoton imaging of ING-2 was performed using a custom-built laser scanning microscope based on a Fluoview300 system (EVIDENT Europe GmbH, Hamburg, Germany), equipped with a 60× water immersion objective (NIR Apo 60×, 1.0 W, Nikon Europe B.V., Amstelveen, the Netherlands). The excitation wavelength was 840 nm; fluorescence emission (filtered with 534/30, catalogue #F39-533, AHF Analysentechnik, Tübingen, Germany) was collected on a phosphomolybdic acid (PMA) hybrid photodetector (PicoQuant GmbH, Berlin, Germany) and registered by a MultiHarp 150 (PicoQuant). The pixel dwell time was 3.81 ms for frames of 512 × 512 pixels and at a typical frame rate of 1 Hz [22]. Wide-field imaging of OGB-1 was performed using an upright microscope (Eclipse FN1, Nikon) equipped with an ORCA FLASH 4.0LT camera (Hamamatsu Photonics Deutschland GmbH, Herrsching am Ammersee, Germany) and a 40× water immersion objective (Fluor 40×/0.8 W, Nikon). Probes were excited using a Polychrome V monochromator (OGB1, ex: 488 nm, em: >505 nm; GCaMP, ex: 488 nm, em: 524–550 nm Thermo Fisher Scientific/FEI, Planegg, Germany) at a framerate of 1 Hz [9].

To induce intracellular ion signals, cellular metabolism was inhibited by perfusing slices with glucose-free ACSF containing 5 mM sodium azide (Riedel de Haen, Selze, Germany) and 2 mM 2-Deoxyglucose (Fluorochem, Hadfield, UK) for 2 min (acute slices) or 1 min (organotypic brain slice cultures), respectively.

## 3. Results

### 3.1. Analysis of Synthetically Generated Data

To assess whether the program can detect the correct number of fluorophore-loaded cells/ROIs, we utilized various sets of synthetically generated data. Figure 1A displays the generated data for multiple objects with their corresponding SNR values, accompanied by the true count of the cells. Each dataset was input into the program and subjected to pre-processing as needed to obtain segmented and labeled images. In addition to identifying cells that remained consistent throughout the stack, the program demonstrated its ability to detect cells appearing later in the image sequence. The cells identified by DL-SCAN are depicted in Figure 1B followed by the corresponding number of detected cells. While certain pre-processing steps aimed at reducing noise in images with low SNR values introduced a disparity between the actual and detected cell counts for some specific datasets (for example, SNR of 0.26), the program’s ability to identify cells from a noisy background remained very good. In cases where a larger number of cells are detected, possibly influenced by the extent of preprocessing steps performed, users have the flexibility to selectively choose the cells of their interest and conduct analyses on those specific selections.

To ensure that the program also detects the correct mean intensity as a function of time and associated parameters, we used distinct single-object datasets with a range of SNR values (Figure 2A). Correspondingly, Figure 2B displays the resultant segmented and labeled images. As observed previously, the program adeptly identifies the ROI in this scenario as well. The mean intensity traces generated by the program closely align with the true traces for all SNR values (Figure 2C).

**Figure 3:**
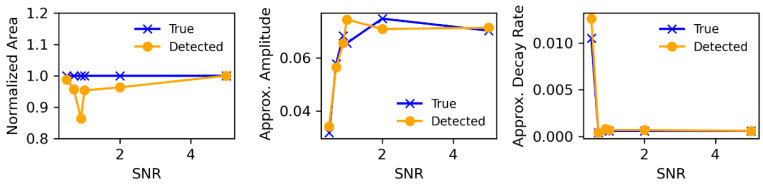
DL-SCAN can accurately extract related parameters from images with different values of SNR. Comparison of the true normalized area, amplitude, and decay rate (s^−1^) with those extracted by DL-SCAN.

We also performed a comparison between the detected cell areas and their true areas with varying SNR, further reinforcing the effectiveness of the program, as shown in Figure 3. However, due to the varying SNR of the images, even minimal preprocessing can affect object edges, particularly in images with low SNR. As a result, the actual total pixel count may differ from the detected pixel count, leading to discrepancies between the two at certain points, as seen in the first plot of Figure 3. Although the program is also capable of computing the rise rate, rise time, decay time, and duration of the obtained trace for each selected cell, here, we only illustrate the decay rate and amplitude for the given dataset for demonstration (Figure 3), which showcases the program’s efficacy. The experiment conducted on the datasets using various additional options available in the program is outlined in the User Manual (Supplementary Information Text).

### 3.2. Application to Experimental Data

The next part of the validation process was implementing DL-SCAN on previously published experiments [9,22]. In this work, acute tissue slices prepared from mouse brains were exposed to inhibitors of glycolysis and oxidative respiration for 2 min to induce chemical ischemia, resulting in transient increases in intracellular ion concentrations in neurons and astrocytes. Experimental data obtained from multiphoton imaging of ING-2 and wide-field imaging of OGB-1 for tracking intracellular Na^+^ and Ca^2+^ in hippocampal CA1 pyramidal neurons and layer II/III cortical astrocytes, respectively, were loaded, segmented, and analyzed. The traces generated using DL-SCAN were then compared to the manually analyzed results.

**Figure 4:**
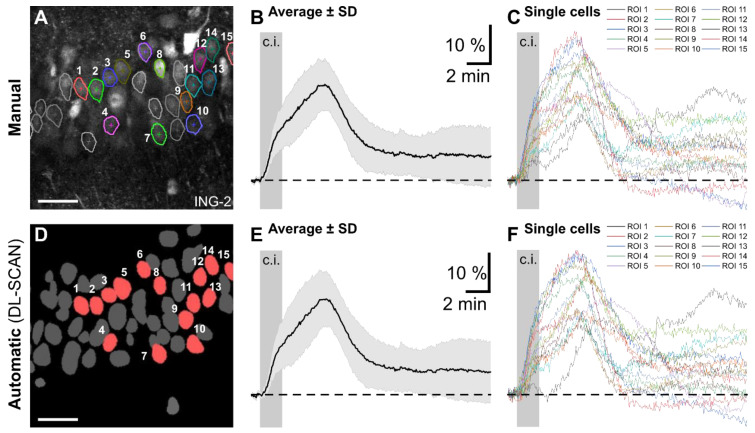
Automated segmentation and analysis by DL-SCAN of fluorescence microscopy imaging of changes in intracellular Na^+^ in hippocampal CA1 pyramidal neurons exposed to a 2-minute period of chemical ischemia. Manual Segmentation using NIS Elements (**A**) and DL-SCAN-segmented neurons on collapsed image (**D**) with average (**B**,**E**) and individual (**C**,**F**) traces for the selected cells. The average intensity trace is represented by the solid line, with the standard deviation shown as error bars in light gray in both (**B**) and (**E**). For accurate comparison, traces from the automated tool were obtained, selecting “No Bleaching Correction” to be consistent with the published manual analysis. Scale: 50 µm. Data taken from [22].

The traces generated using DL-SCAN were then compared to the manually analyzed results. As shown in Figure 4, the changes in ING-2 fluorescence for the selected ROIs, representing neuronal cell bodies in the CA1 pyramidal cell layer, are almost identical. The minor discrepancies are most likely due to minimal differences in the ROI placement in the manual segmentation of the cells in the top panel of Figure 4A versus their automated identification as shown in Figure 4D. However, these variations are minor and not biologically relevant compared to the signal-to-noise ratio in these experiments.

**Figure 5:**
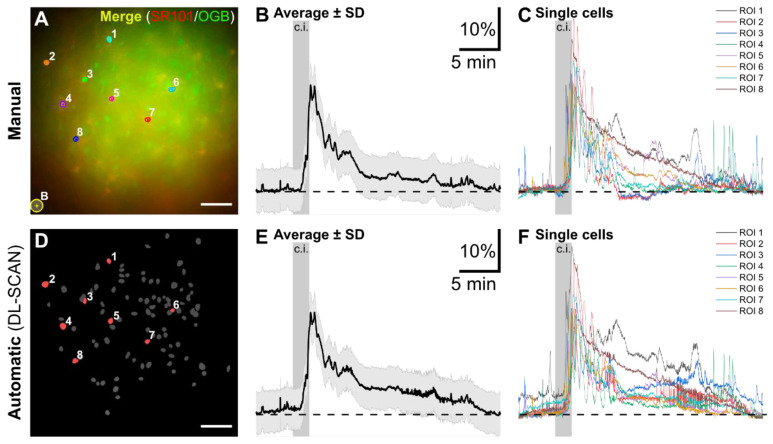
Automated segmentation and analysis by DL-SCAN of fluorescence microscopy imaging of changes in intracellular Ca^2+^ using the chemical indicator OGB in layer II/IIII cortical astrocytes exposed to a 2-minute period of chemical ischemia. Manual Segmentation using NIS Elements (**A**) and DL-SCAN-segmented astrocytes (**D**) with average (**B**,**E**) and individual traces (**C**,**F**) for the selected cells. The average intensity trace is represented by the solid line, with the standard deviation shown as error bars in light gray in both (**B**) and (**E**).

In addition, we reanalyzed and compared changes in intracellular Ca^2+^ induced by chemical ischemia as measured by the chemical indicator OGB in astrocytes in cortical brain slices [9]. The changes in astrocytic OGB-1 fluorescence for manual and automatic segmentation are shown in Figure 5A,D. This part of the analysis was performed using the bleaching correction option. In line with the manual analysis published previously [9], the initial and final 10 frames were utilized for interpolating intermediate values. These values were subsequently subtracted from the original trace to correct for photobleaching. The normalized results obtained clearly demonstrate the close correspondence between the traces obtained from manual analysis and from DL-SCAN. The elimination of the negative values from the latter was performed by subtracting the absolute minimum value from all intensity values. Although this explains the difference in the traces as the normalized intensity values drop below 0 in manual analysis, it has no significant effect on the subsequent analysis.

**Figure 6:**
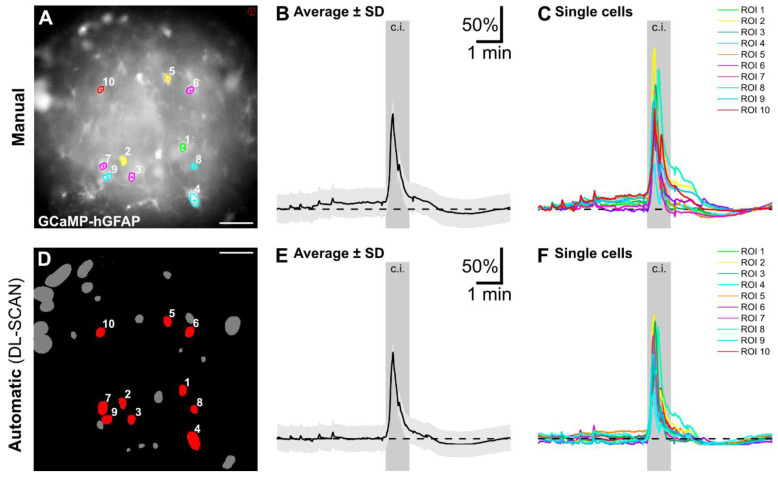
Automated segmentation and analysis by DL-SCAN of fluorescence microscopy imaging of changes in intracellular Ca^2+^ using the genetically encoded indicator in GCaMP in layer II/III cortical astrocytes exposed to a 1-minute period of chemical ischemia. Manual Segmentation using NIS Elements (**A**) and DL-SCAN-segmented astrocytes (**D**) with average (**B**,**E**) and individual traces (**C**,**F**) for the selected cells. The average intensity trace is represented by the solid line, with the standard deviation shown as error bars in light gray in both (**B**) and (**E**).

As a third proof of principle, we reanalyzed and compared changes in the intracellular Ca^2+^ concentration induced by a 1-minute period of chemical ischemia in astrocytes using the genetically encoded Ca^2+^ indicator GCaMP expressed under the GFAP promotor in organotypic slices of the mouse cortex. The manually and automatically collected data are shown in Figure 6. For bleaching correction, the first and last 30 frames of the measurement were used. The normalized traces of both types of analysis show a very similar time course, underlying previous results indicating that the manual and automatic data collection by DL-SCAN have a high correspondence.

**Figure 7:**
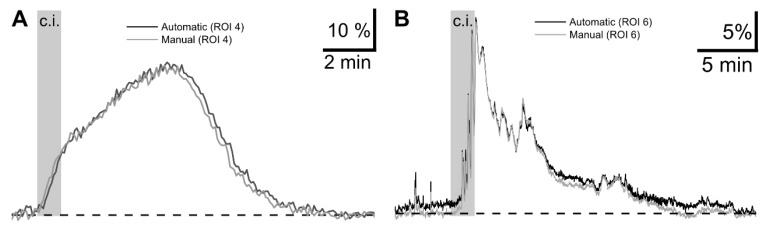
Time traces extracted by DL-SCAN show that the program accurately detects the temporal evolution of the signal. Direct comparison between manually and DL-SCAN-extracted traces (**A**) of changes in Na^+^ in a randomly selected hippocampal neuron and (**B**) in Ca^2+^ in a randomly selected cortical astrocyte exposed to brief chemical ischemia.

Direct comparison between the manual and DL-SCAN analyses for each experiment (neuronal Na^+^ imaging and astrocytic Ca^2+^ imaging), shown in Figure 7A,B for two of the ROIs, also validates the efficacy of the program.

**Figure 8:**
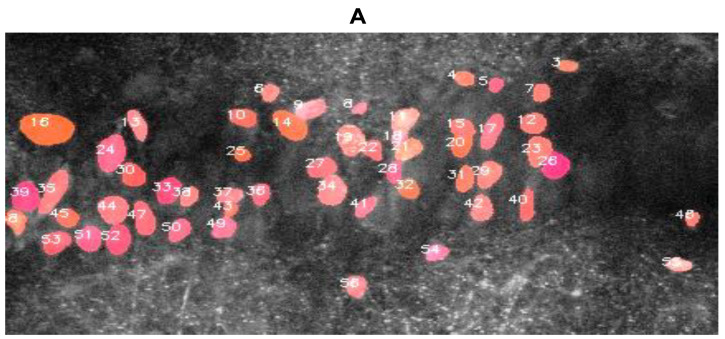

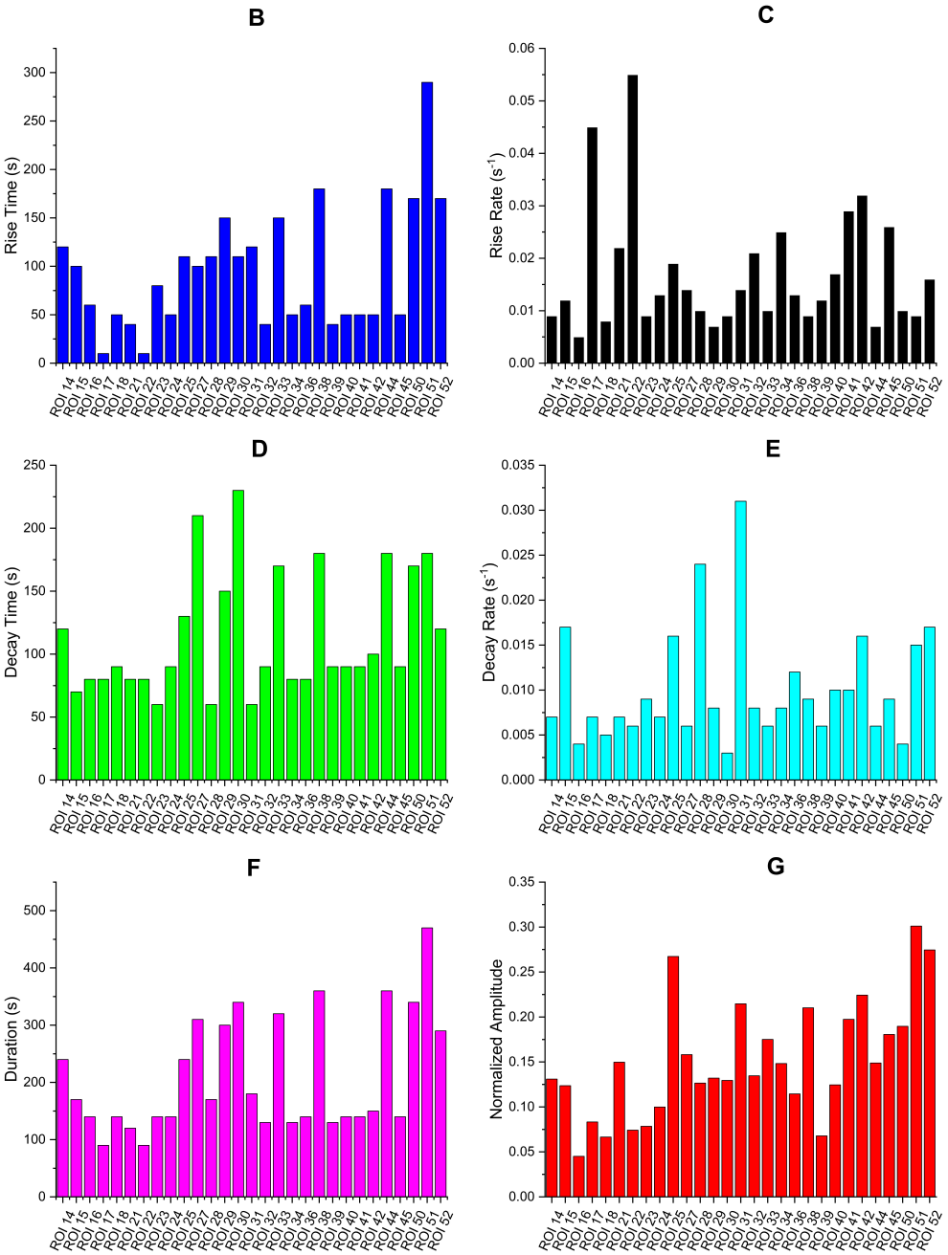
DL-SCAN provides analysis of relevant parameters on a cell-by-cell basis. (**A**) Automatically segmented and labeled neurons in an ING-2 loaded hippocampal tissue slice. Bar plots show properties of the transient changes in intracellular fluorescence upon brief chemical ischemia: rise time ((**B**), mean ± SD, 95 ± 63 s), rise rate ((**C**), 0.017 ± 0.012 s^−1^), decay time ((**D**), 114 ± 48 s), decay rate ((**E**), 0.010 ± 0.006 s^−1^), duration ((**F**), 209 ± 101 s), and normalized amplitude ((**G**), 0.15 ± 0.06) for the selected cells using DL-SCAN. Each bar in panels (**B**–**G**) corresponds to a single cell.

In Figure 8, we show DL-SCAN’s ability to generate various statistics for the selected ROIs from the segmented objects in another experiment in which changes in ING-2 fluorescence induced by chemical ischemia were measured in CA1 pyramidal neurons [22]. Contingent upon various post-processing options like baseline estimation, photobleaching correction, static or dynamic analysis, etc., relevant information about the rise time, rise rate, decay time, decay rate, duration, and amplitude of time traces can be generated for user-selected cells. These quantities provide instant, valuable data on the recorded signals in selected cells. For instance, the accumulation and clearance of ions in specific cellular compartments, as well as the comparison of the extent of ion fluxes across different experimental conditions or cell types, can be quantified using these parameters. DL-SCAN provides a very efficient and user-friendly way to perform such automated analysis. The statistics for the selected neurons that are shown in Figure 8 were generated by selecting the “Dynamic” analysis option, with the baseline estimated as the average of the first 10 frames. The program provides bar plots of these parameters for each cell (Figure 8). The relevant data can also be exported in ascii format for further analysis and visualization.

As mentioned above, the program also generates histograms of decay rates, rise rates, decay times, rise times, durations, and normalized amplitudes of traces from all cells detected in the image stack. In Figure S16, we show these distributions for cells detected in Figure 8A.

### 3.3. Quantification of Morphological Changes and Temporal Correlation in Cells

**Figure 9:**
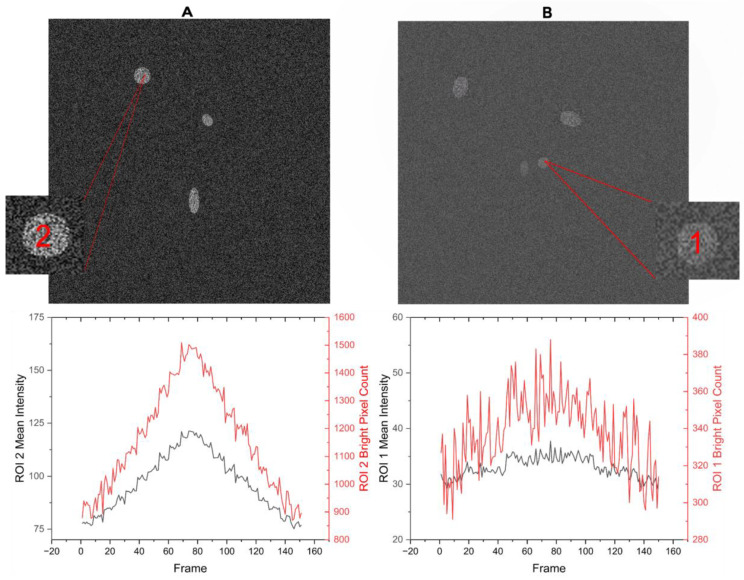
DL-SCAN can track the temporal changes in the area of cells in the image stack. DL-SCAN segmented synthetically generated cells (**A**,**B**) (upper panels). Examples of the change in the cell’s area (bright pixel count) and mean intensity for the generated synthetic data (**A**,**B**) (lower panels).

Next, we demonstrate the tool’s ability to account for the morphological changes in cells, with the help of synthetic data. Preferably, quantifying cell swelling or shrinking requires a specific cell-permeant dye like Calcein-AM that diffuses across cytosol and remains inside the cell for longer period, emitting green light under blue light excitation [27,28]. The longer retention time then becomes useful in monitoring the changes in cell area over time. To demonstrate this ability, we employ synthetic data generated to represent swelling in a cell stained with a Calcein-like dye by gradually increasing the size of the object and decreasing it again to normal over time. The corresponding results are shown in Figure 9. The counts of bright pixels (pixels exceeding the threshold multiplied by the maximum pixel value) are obtained using the default area percentage threshold of 0.3. This essentially refers to the count of pixels within each ROI that exceed 30% of the maximum pixel value. Figure 9 effectively estimates the change in the morphology of cells over time when segmented on the collapsed image, demonstrating the ability of DL-SCAN to track changes in cell area. We remark that this is an indirect method, which, in a real-life scenario, could be influenced by the uptake, adsorption, and dilution of the fluorophores. Therefore, we recommend careful evaluation of the results in future use of the tool for analyzing cell swelling.

DL-SCAN also calculates temporal correlation between the fluorescence changes in all cells in the image stack, enabling the user to look at the propagation of signal from one cell to another in case insights into the propagation of intercellular wave are desired (Figure S15). The centroid position for each selected ROI can be displayed followed by the cell correlation matrix and a heatmap. By identifying the positions of these ROIs, one can track how signals, such as changes in fluorescence, travel between cells. This approach could be used to investigate cell-to-cell communication, including the propagation of electrical or chemical signals.

## 4. Discussion 

Existing microscope manufacturer tools like NIS Elements by Nikon Instruments or CellSense FluoView by Evident Europe primarily require manual selection of ROIs for analysis. The reliance on this manual labeling and analysis is time-consuming and subjective, particularly when working with large datasets or complex images. Automated tools like AQuA (Astrocyte Quantification and Analysis), CaSCaDe (Ca^2+^ Signal Classification and Decoding), and MSparkles identify ROIs based on changes in intensity of neighboring pixels [14,15,16]. Instead of picking specific regions to study beforehand, AQuA uses the whole image to find patterns in how fluorescence changes over time, while MSparkles uses a region-growing approach to identify ROIs [14,15]. While tools like these offer flexibility in terms of algorithm customization and parameter tuning, which can be beneficial for researchers with specific needs or for analyzing diverse datasets, convolutional neural network (CNN)-based approaches prove to be very effective in terms of accuracy even for generalizing variable image patterns and tasks like ROI identification. The various components of CNN architecture, such as convolutional layers, pooling layers, skip connections, regularization layers, loss functions and activation functions, work together to extract and preserve complex features, spatial information and details from the input image in an unprecedented manner. One such tool with a multitude of options for the analysis of fluorescence calcium imaging data obtained from experiments using calcium-sensitive dyes or genetically encoded calcium indicators (GECIs) is CaImAn [29]. While it is an extremely powerful tool that performs tasks like fluorescence signal extraction and motion correction in real time as data are being collected, its algorithm is primarily designed and optimized for calcium dynamics. This may limit its flexibility for use with other ions like Na^+^.

To overcome the shortcomings in the existing approaches mentioned above, we developed DL-SCAN, which is user-friendly yet equally effective for cell segmentation and analysis from various imaging experiments. The higher Average Precision (AP) cell segmentation accuracy of StarDist as compared to other popular object segmentation models for various intersection over union threshold values proves to be a significant advancement in the field of automated cell segmentation [17]. Its performance showcases its robustness and accuracy in delineating cell boundaries, which is crucial for biological applications. Accurate cell identification helps immensely in correct quantification of cellular features, cellular functions, cellular localization, and cellular interactions. It is also essential for visualizing and analyzing cells in tissues, which is critical for identifying and monitoring the diseased state. Therefore, we decided to incorporate StarDist into our tool, resulting in high-quality cell segmentation outcomes. This was followed by various user-adjustable options to facilitate and streamline efficient analysis as needed by generating relevant statistics. Although DL-SCAN generates publication-quality figures incorporating a comprehensive analysis of the data, options are provided to download the generated files in CSV format to enable users to load the data to external software for further processing, analysis, and visualization. We believe that this tool will benefit researchers and experimentalists in the imaging field to study cells and/or cellular compartments by significantly reducing time consumption and bias while increasing accuracy.

Synthetic data with varying SNR, which were representative of cellular somata (neurons and astrocytes), were generated to test the accuracy of the cell segmentation and their statistics. Secondly, the tool was tested on Na^+^ imaging data from hippocampal neurons, Ca^2+^ imaging data from cortical astrocytes as published previously [9,22], and Ca^2+^ imaging data using a genetically encoded indicator expressed in astrocytes in organotypic brain slice cultures. The outcomes were then compared to the previously published analyses performed using NIS Elements AR 5.0 software from Nikon instruments [9,22]. The time it took to find the best settings depended on the preparation and experimental setup. In our case, the experimentalists could use the standard settings of the program for experiments illustrated in Figure 4, which means there was essentially no additional time involved. As opposed to the time-consuming manual analysis performed before, DL-SCAN easily and rapidly managed to automatically segment somatic ROIs. For experiments illustrated in Figure 4, Figure 5 and Figure 6, it took approximately 2–3 h to find parameters that (a) represented physiological cell boundaries, (b) confirmed that the cell boundaries were virtually identical to regions that would have been drawn manually, and (c) confirmed that the traces were identical to those obtained from manual analysis. This initial time required significantly decreased with repeated use and experience of the user. Of note, it was still significantly less than the time required for manual analysis. The time savings are, therefore, a substantial benefit of DL-SCAN. Furthermore, DL-SCAN enabled immediate extraction of their fluorescence signals over time as well as of other parameters, also offering numerous user-adjustable options for pre-processing, and analysis options related to the specific experimental setup. These features help to delineate and objectivize the entire image analysis process.

Along with intensity changes over time, this tool also records bright pixel counts above a certain user-specified threshold for each detected cell. Since cells can be segmented based on the first or the collapsed image, the “Area Threshold Percentage” option is introduced to account for cell swelling when segmentation is performed on the latter. The pixel count of a region greater than the threshold percentage of the maximum pixel value for that region is generated and plotted. This helps to track the changes in the area of the identified cell over time, as illustrated earlier, but likely has some limitations. This feature of the tool can, for example, be used with a fluorescent indicator that is inert to changes in ion concentrations and does not respond to other cellular signals. For dyes that become brighter when they are binding to ions as the signal spreads spatially inside the cell (e.g., the propagation of a Ca^2+^ wave in the cytoplasm), recording the pixel count surpassing a certain brightness threshold may not work. Nevertheless, the algorithm will accurately track changes in the cell area in images where the ROI is changing due to cell swelling. One of the other limitations in cell detection occurs when segmenting images with a very low SNR. As seen in the third image of Figure 1, extensive preprocessing to make such images segmentation-ready can sometimes cause the algorithm to detect regions that do not actually correspond to cells. However, since users can select specific detected ROIs for further analysis, this limitation can be addressed. Still, when heavy preprocessing alters object edges, the true area of the ROI may not be accurately recovered, as seen in certain low-SNR regions in Figure 3. This can lead to inaccurate cell analysis, particularly in images with low SNR. Nevertheless, the SNRs at which DL-SCAN fails are much smaller than those of the experimental tools used in such experiments.

## 5. Conclusions

To summarize, the major benefit of DL-SCAN is its ease of use with a wide variety of datasets, its accuracy, avoiding user-bias as the deep learning algorithm automatically segments and analyzes the cells, and its access to additional features and statistics that are not incorporated in other tools. Although tailored for a certain use case (acute brain slices), the nature of the detection algorithm and the subsequent analysis options, together, make it easily adaptable for a broad range of applications. Furthermore, our open-source algorithm provides an opportunity to update and customize the application as needed.

## Data Availability

All data is included in the paper. The source code for the software is available in the GitHub repository at https://github.com/banalok/dlscan.

## Supplementary Materials

The following supporting information can be downloaded at https://www.mdpi.com/article/10.3390/biom14111348/s1: Supplementary Text: User manual and step-by-step instructions for using DL-SCAN.
